# The Accoucheur’s Emergency Manual

**Published:** 1895-04

**Authors:** 


The Accoucheur's Emergency Manual. By W. A. Yingling, M. D., Ph. D., member of the International Hahnemannian Association, Philadelphia: Boericke & Tafel. 1895. Flexible leather. Price, $1.25 net; by mail, $1.30.

This beautiful little duodecimo of 324 pages contains indications of all the remedies that are most useful in the treatment of women in confinement.

The book is in two parts. Part first contains the remedies in alphabetical order, with their most prominent indications, and part second contains the Repertories.

These Repertories are as follows: Labor, Abortion, Hemorrhage, Retained Placenta, Convulsions, After-pains, and the Baby.

That the book is in the line of pure Hahnemannian Homœopathy, is its pre-eminently valuable feature, especially in these days when such quantities of books of mixed practice are being showered from the press.

The name of the author is a sufficient guarantee that the book would be strictly homœopathic. He is well known to the readers of THE HOMŒOPATHIC PHYSICIAN for his many valuable articles upon homœopathic subjects which have appeared in its pages from time to time.

In his introduction the author says: "The only direction that can be given for prescribing is to follow the direction of Samuel Hahnemann, as given in *The Organon* and *The Chronic Diseases*. Discard every fad, every allopathic adjunct, and rely implictly on the homœopathic remedy. Live or die true to your declared principles. The female eunuchs, the female invalids, and female maniacs resulting directly from allopathic means of meddlesome midwifery should deter any follower of Hahnemann from resorting to their means of treatment"

The readers of this journal will peruse with pleasure this refreshing declaration of sturdy homœopathic principle, and be abundantly satisfied of the orthodox character of the book.

The editor is tempted to yet many more quotations from this book, but forbears for want of space.

In conclusion it may be said the book is beautifully printed in clear type, of small size, for the pocket, and has within it only such information as is absolutely necessary at the bedside, and so arranged as to be ready for immediate reference.

